# Assessment of Insulin Therapy as a Risk Factor for Hirsutism Among Diabetic Females in Saudi Arabia

**DOI:** 10.7759/cureus.32512

**Published:** 2022-12-14

**Authors:** Abdullah J Alghanim, Faisal M Alfalah, Ali R Al Zaid, Mohammed AlRamadan, Khalid A Alhuwayji, Hussain M Alnasser, Ali T Alamer, Suha Albahrani

**Affiliations:** 1 Medicine, King Faisal University, Al-Ahsa, SAU; 2 Family Medicine, King Faisal University, Al-Ahsa, SAU

**Keywords:** insulin therapy, insulin intake, diabetes mellitus type 1, diabetes mellitus type 2, insulin resistance, hirsutism

## Abstract

Introduction

Hirsutism is defined as a condition in which women develop excessive body hair in androgen-dependent areas, which include lips, chin, chest, abdomen, back, and femoral region. The link between hyperandrogenism and insulin resistance and/or hyperinsulinemia is well established. Polycystic ovary syndrome, as a form of hyperandrogenism, has been linked to several diseases, including type 2 diabetes mellitus and hirsutism. However, it is unknown how common hyperandrogenic problems are in women who receive exogenous insulin. Therefore, this study aims to assess the effect of insulin intake and other sociodemographic factors on the development of hirsutism among diabetic females.

Methods

This case-control study was conducted in six regions of Saudi Arabia, including Al-Ahsa, Dammam, Qatif, Riyadh, Abha, and Jeddah, during the year 2022. The population was Saudi females who were diabetic, between the age of 18 and 65 years, and living in Saudi Arabia. The sample size was 186 participants. Of the participants, 48 had considerable hirsutism whereas 138 did not. The degree of hirsutism has been determined using the Ferriman and Gallwey scoring tool.

Results

A total of 186 diabetic females were included in the study. Among the females, 97 (52.2%) were on insulin therapy and 89 (47.8%) were on non-insulin therapies. Only hair distribution on the chin showed a significant difference between the study groups where 4.1% of cases on insulin showed complete cover with light or heavy hair on the chin compared to 3.4% of controls (P = 0.049). There was no significant difference regarding hirsutism score among the study patients according to insulin intake where the mean score was 5.4 ± 5.1 among cases on insulin versus 4.7 ± 5.1 for controls (P = 0.978). Adjusted logistic regression models showed an insignificant association between diabetic female hirsutism and insulin intake (OR = 1.1 and 1.0, respectively; P > 0.05).

Conclusion

Many factors were examined to reveal their associations with hirsutism in diabetic females. Neither the type of diabetes nor insulin intake was significantly correlated with the development of hirsutism. On the other hand, age was found to be significantly associated with the development of hirsutism among age groups (<30, 30-49, and 50+; P = 0.49). It seemed that the prevalence of hirsutism decreases as age advances.

## Introduction

Type 1 diabetes mellitus (T1DM) is an autoimmune disease that destroys endocrine pancreatic B-cells, resulting in insulin insufficiency, which leads to a hyperglycemic state [1]. T1DM usually begins in childhood or adolescence and lasts for the rest of the person's life [1]. A study done in 2010 showed that the prevalence of T1DM worldwide is around 285 million [1]. In 2007, a study revealed that the prevalence of T1DM in Saudi Arabia among children and adolescents was 109.5 per 100,000 [2]. Type 2 diabetes mellitus (T2DM), on the other hand, is believed to be caused by two distinct pathological mechanisms [1]. First, there is altered insulin secretion as a result of pancreatic beta-cell dysfunction. Second, insulin action is altered through tissue insulin resistance [1]. Furthermore, Kaiser et al. estimated the global prevalence of T2DM to be 500 million in 2018. From a local perspective, the overall prevalence was estimated to be 32.8% in 2015 and is predicted to reach 45.36% in 2025 [3].

Insulin is a peptide hormone produced normally by the human body to control blood glucose levels [4]. However, in the case of diabetes mellitus (DM), the body either cannot produce insulin or has resistance to it, so an external source of insulin needs to be used daily and is usually injected subcutaneously to treat T1DM or the advanced cases of T2DM [4].

However, even though insulin therapy is necessary to treat T1DM and might be needed for T2DM, concerns about the safety and side effects of insulin therapy remain active [5]. The side effects of insulin therapy range from mild to severe, including hypoglycemia, weight gain, insulin allergic reaction, and lipoatrophy or lipohypertrophy at the site of injection [5].

There is a well-established link between hyperandrogenism and insulin resistance and/or hyperinsulinemia problems [6]. Polycystic ovary syndrome (PCOS) has been linked to a number of medical conditions that include insulin resistance, including T2DM [6,7]. However, it is uncertain how common hyperandrogenic problems are in women with T1DM [6].

The use of exogenous insulin to treat DM in those patients has been thought to have contributed to the development of PCOS. Also, since hyperinsulinemia and hyperandrogenemia are closely related, obese women and those with T1DM are likely to have a higher prevalence of hirsutism, which is defined as a condition in which women develop excessive body hair in androgen-dependent areas and menstrual problems than the general female population [8,9]. In a study conducted on 174 girls, 78 of them were diagnosed with T1DM, and 16 (21%) out of those had hirsutism [8].

There are lots of other risk factors associated with hirsutism, including smoking, certain drugs (oral contraceptives, L-thyroxine, danazol, and diazoxide), and certain syndromes (PCOS, obesity, insulin resistance, hyperprolactinemia, hyperthecosis, congenital adrenal hyperplasia, and idiopathic hirsutism) [10,11].

A study conducted on 30 patients showed the major risk factor associated with hirsutism is PCOS (53.33%). Hirsutism was found in 40% of the patients aged between 14 and 22 years, 30% aged up to 45 years, and 30% aged more than 50 years. Around 43% of patients had a family history of hirsutism. Premature adrenarche was found in 10% of patients. Women having hirsutism after menopause were 6.66%. About 60% of women were having irregular menstruation, and 20% of women were overweight [10].

In this study, we aim to assess insulin therapy as a risk factor for hirsutism among women with T1DM and T2DM in Saudi Arabia. Also, it aims to establish a new database about the relationship between hirsutism and the other risk factors in females with DM.

## Materials and methods

Study design and participants

A case-control study was conducted in six regions of Saudi Arabia, including Al-Ahsa, Dammam, Qatif, Riyadh, Abha, and Jeddah, during the year 2022. The population was Saudi females who were diabetic, between the age of 18 and 65 years, and living in Saudi Arabia. All types of diabetes were included in the study. The sample size was 186 participants. Of the participants, 48 had considerable hirsutism whereas 138 did not. The degree of hirsutism has been determined using the Ferriman and Gallwey scoring tool [12]. The participants were recruited via two methods, which were based on the convenience sampling technique. First, most of the participants were recruited online after adopting their contact numbers from diabetes clinics. Second, many participants were recruited and invited to participate in an in-person encounter at a diabetes center in Al-Ahsa, Saudi Arabia.

Data analysis

The data were collected, reviewed, and then fed to Statistical Package for Social Sciences version 21 (IBM Corp., Armonk, NY). All statistical methods used were two-tailed with an alpha level of 0.05 considering significance if the P-value was less than or equal to 0.05. Visual methods to determine the degree of hirsutism, as originally described by Ferriman and Gallwey, were used [12]. The density of terminal hairs at different body sites (lips, chin, chest, upper abdomen, lower abdomen, upper arm, forearm, thigh, lower leg, upper back, and lower back) was scored from 0 to 4, and the total score was calculated. The researchers assessed each participant individually based on the scoring assessment of the Ferriman-Gallwey scoring system. A score of 8 or more was considered significant and diagnostic for hirsutism while a score of less than 8 was considered non-significant. Descriptive analysis was done by prescribing frequency distribution and percentage for study variables including patient's biodemographic data, BMI, and medical history of PCOS and epilepsy, besides diabetes data. Cross-tabulation was used to assess hirsutism among study diabetic females according to insulin therapy. To assess the distribution of hirsutism among diabetic females by their biodemographic data, the Pearson chi-square test for significance was used and the exact probability test was used if there were small frequency distributions. Univariate logistic regression was used to assess the crude association between insulin therapy and hirsutism among diabetic females, and then a hierarchical logistic model was used to adjust for the effect of other factors including patients' age, BMI, type of DM, duration of DM, and PCOS.

## Results

A total of 186 diabetic females were included in the study (Table 1). Among the females, 97 (52.2%) were on insulin therapy and 89 (47.8%) were on non-insulin therapies. The ages of the patients ranged from 12 to 75 years with a mean age of 45.3 ± 13.7 years. The duration of diabetes ranged among cases from less than one year to 45 years with a mean duration of DM of 12.5 ± 9.6 years. Around 38% (71) of the patients were obese, and 37.1% (69) were overweight. Among the patients, 77 (41.4%) had T1DM and 109 (58.6%) had T2DM. The number of PCOS cases in the study was 30 (16.1%), and 66 (35.5%) had undergone permanent hair removal.

**Table 1:** Biodemographic data of diabetic female patients on insulin (cases) and others with no insulin (controls) DM: diabetes mellitus.

Biodemographics	Patients		
			No	%	
	Age in years			
<30	32	17.2%	
30-49	65	34.9%	
50+	89	47.8%	
Duration of DM in years			
<5	41	22.0%	
5-10	51	27.4%	
>10	94	50.5%	
Body mass index			
Normal weight	46	24.7%	
Overweight	69	37.1%	
Obese	71	38.2%	
Type of DM			
Type 1 DM	77	41.4%	
Type 2 DM	109	58.6%	
Had polycystic ovarian syndrome			
Yes	30	16.1%	
No	156	83.9%	
Complained of epilepsy			
Yes	1	.5%	
No	185	99.5%	
Have you undergone any type of permanent hair removal to get rid of your body hair?			
Yes	66	35.5%	
No	120	64.5%	

Only hair distribution on the chin showed a significant difference between the study groups where 4.1% of cases on insulin showed complete cover with light or heavy hair on the chin compared to 3.4% of controls (P = 0.049). Regarding the upper lip, a complete lack of terminal hair was detected among 54.6% of cases on insulin versus 47.2% of controls (P = 0.187). Also, there was no significant difference regarding hirsutism in other body locations between the two groups (Table 2).

**Table 2:** Hirsutism among study diabetic females according to insulin therapy P: Pearson X2 test; $: exact probability test; * P < 0.05 (significant).

Site		Total		Group				P-value
				Insulin cases		Controls		
		No	%	No	%	No	%	
Upper lip	Complete lack of terminal hairs	95	51.1%	53	54.6%	42	47.2%	0.187^$^
	A small number of terminal hairs over the upper lip and outer lip border	62	33.3%	35	36.1%	27	30.3%	
	Thin mustache covering less than 50% of the upper lip or at the outer border	20	10.8%	6	6.2%	14	15.7%	
	Mustache covering 50% of the outer margin of the lip or 50% of the lip height	6	3.2%	2	2.1%	4	4.5%	
	Mustache covering most of the upper lip and crossing the midline lip	3	1.6%	1	1.0%	2	2.2%	
Chin	Complete lack of terminal hairs	118	63.4%	70	72.2%	48	53.9%	0.049*^$^
	Few scattered hairs	46	24.7%	19	19.6%	27	30.3%	
	Scattered hairs with small concentrations	15	8.1%	4	4.1%	11	12.4%	
	Complete cover with light hair	6	3.2%	3	3.1%	3	3.4%	
	Complete cover with heavy hair	1	0.5%	1	1.0%	0	0.0%	
Upper arms	Complete lack of terminal hairs	132	71.0%	70	72.2%	62	69.7%	0.375
	Sparse growth affecting not more than one-quarter of the limb surface	39	21.0%	17	17.5%	22	24.7%	
	Increased, but incomplete coverage	9	4.8%	5	5.2%	4	4.5%	
	The entire area is covered with a light growth	3	1.6%	3	3.1%	0	0.0%	
	The entire area is covered with a heavy growth	3	1.6%	2	2.1%	1	1.1%	
Chest	Complete lack of terminal hairs	137	73.7%	71	73.2%	66	74.2%	0.317^$^
	Circumareolar or midline terminal hairs	40	21.5%	22	22.7%	18	20.2%	
	Circumareolar and midline terminal hairs	7	3.8%	2	2.1%	5	5.6%	
	75% of the chest covered with terminal hairs	2	1.1%	2	2.1%	0	0.0%	
	The entire area is covered with terminal hair growth	0	0.0%	0	0.0%	0	0.0%	
Upper abdomen	Complete lack of terminal hairs	126	67.7%	64	66.0%	62	69.7%	0.581^$^
	Scattered midline terminal hairs	42	22.6%	22	22.7%	20	22.5%	
	More terminal, still midline	13	7.0%	7	7.2%	6	6.7%	
	50% of the upper abdomen covered	3	1.6%	3	3.1%	0	0.0%	
	The entire area is covered with terminal hair growth	2	1.1%	1	1.0%	1	1.1%	
Lower abdomen and pubic hair	Complete lack of terminal hairs	61	32.8%	30	30.9%	31	34.8%	0.601
	A small number of scattered midline terminal hairs at the length of the linea alba	66	35.5%	39	40.2%	27	30.3%	
	Midline concentration of terminal hair at the length of the linea alba	24	12.9%	11	11.3%	13	14.6%	
	A midline thickened band of terminal hair less than ½ width of pubic hair at the base	16	8.6%	9	9.3%	7	7.9%	
	An inverted V-shaped coverage of 2 widths of pubic hair at the base	19	10.2%	8	8.2%	11	12.4%	
Upper thighs	Complete lack of terminal hairs	93	50.0%	48	49.5%	45	50.6%	0.211
	Scattered midline terminal hairs	54	29.0%	23	23.7%	31	34.8%	
	More terminal, still midline	24	12.9%	15	15.5%	9	10.1%	
	50% of the upper abdomen covered	10	5.4%	7	7.2%	3	3.4%	
	The entire area is covered with terminal hair growth	5	2.7%	4	4.1%	1	1.1%	
Upper back	Complete lack of terminal hairs	139	74.7%	73	75.3%	66	74.2%	0.320^$^
	Sparse terminal hairs over the upper back	37	19.9%	16	16.5%	21	23.6%	
	Increased number of spread terminal hairs	8	4.3%	6	6.2%	2	2.2%	
	The entire area is covered with a light growth	1	0.5%	1	1.0%	0	0.0%	
	The entire area is covered with a heavy growth	1	0.5%	1	1.0%	0	0.0%	
Lower back\sacral	Complete lack of terminal hairs	151	81.2%	77	79.4%	74	83.1%	0.757^$^
	Sacral area with hair coverage less than 4 cm wide	24	12.9%	13	13.4%	11	12.4%	
	Increased side coverage	6	3.2%	3	3.1%	3	3.4%	
	75% of the lower back is covered with terminal hairs	4	2.2%	3	3.1%	1	1.1%	
	The entire area is covered with heavy growth	1	.5%	1	1.0%	0	0.0%	

**Figure 1:**
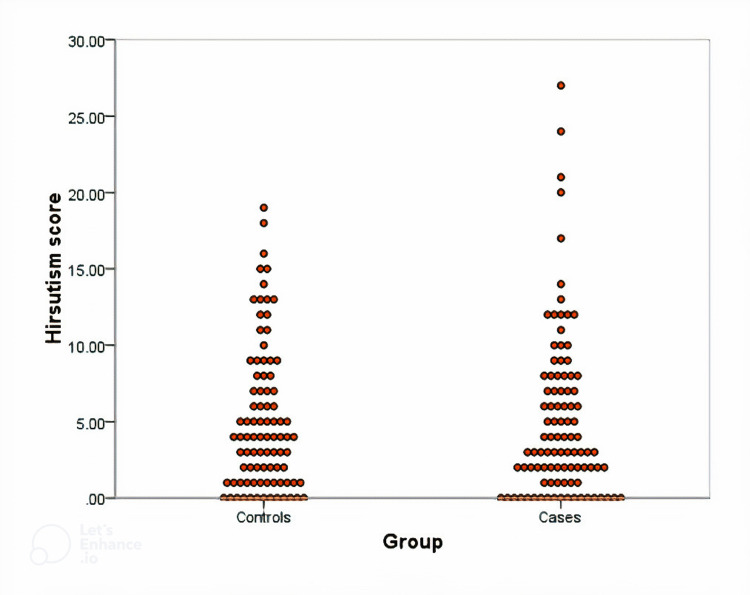
Hirsutism score among study groups of diabetic females

There was no significant difference regarding hirsutism score among the study participants and insulin intake where the mean score was 5.4 ± 5.1 among cases on insulin versus 4.7 ± 5.1 for controls (P = 0.978), as shown in Figure 1.

**Figure 2:**
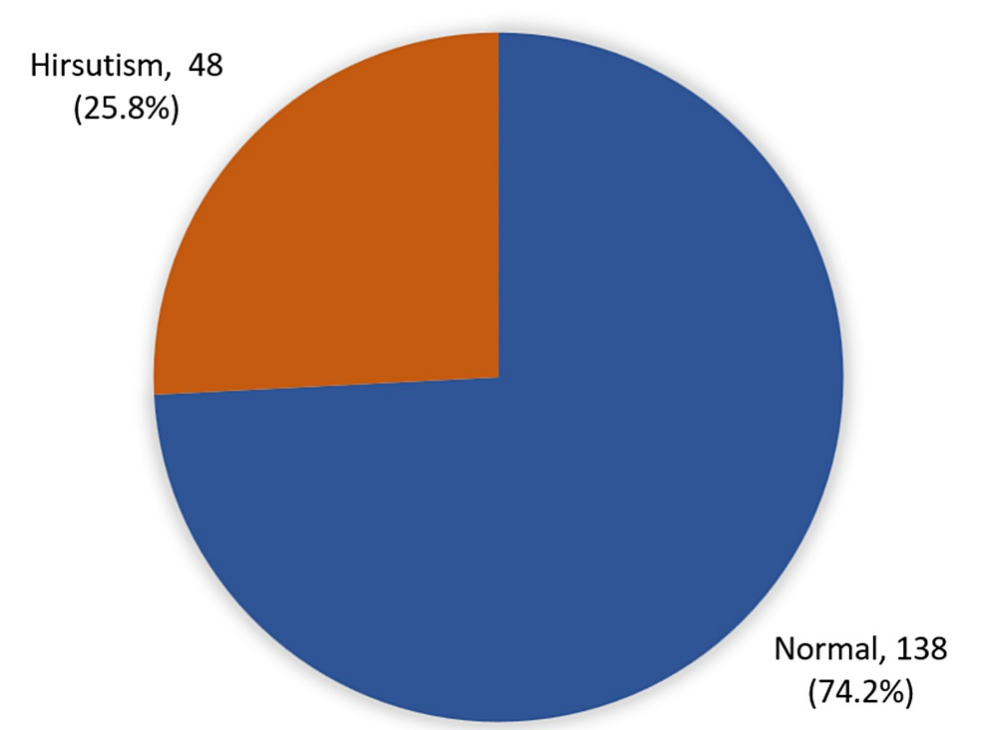
Overall prevalence of hirsutism among the study diabetic females

The overall prevalence of hirsutism among diabetic females is illustrated in Figure 2. Precisely, 48 (25.8%) diabetic females had considerable hirsutism whereas 138 (74.2%) did not have hirsutism.

Table 3 correlates the distribution of hirsutism among diabetic females with their biodemographic data. Hirsutism was detected among 37.5% of young aged diabetic cases compared to 18% of others aged 50 years or more with recorded statistical significance (P = 0.049). Also, 40.9% of cases who had undergone permanent hair removal had hirsutism versus 17.5% of others who did not have permanent hair (P = 0.001).

**Table 3:** Distribution of hirsutism among diabetic females by their biodemographic data P: Pearson X2 test; * P < 0.05 (significant); DM: diabetes mellitus.

Biodemographic data	Hirsutism				P-value
	No		Yes		
	No	%	No	%	
Age in years					0.049*
<30	20	62.5%	12	37.5%	
30-49	45	69.2%	20	30.8%	
50+	73	82.0%	16	18.0%	
Type of DM					0.469
Type 1 DM	55	71.4%	22	28.6%	
Type 2 DM	83	76.1%	26	23.9%	
Duration of DM in years					0.129
<5	26	63.4%	15	36.6%	
5-10	37	72.5%	14	27.5%	
>10	75	79.8%	19	20.2%	
Body mass index					0.132
Normal weight	29	63.0%	17	37.0%	
Overweight	53	76.8%	16	23.2%	
Obese	56	78.9%	15	21.1%	
Had polycystic ovarian syndrome					0.138
Yes	19	63.3%	11	36.7%	
No	119	76.3%	37	23.7%	
Have you undergone any type of permanent hair removal to get rid of your body hair?					0.001*
Yes	39	59.1%	27	40.9%	
No	99	82.5%	21	17.5%	

Table 4 shows an insignificant association between diabetic female hirsutism and insulin intake (OR = 1.1 and 1.0, respectively; P > 0.05).

**Table 4:** Univariate and adjusted logistic regression analysis for associating between diabetic females' hirsutism and intake of insulin OR_C_: crude odds ratio; OR_A_: adjusted odds ratio for patients' age, type and duration of DM, PCOS, and BMI; CI: confidence interval; DM: diabetes mellitus; PCOS: polycystic ovary syndrome.

Group	Hirsutism				P-value	OR_C_ (95% CI)	OR_A _(95% CI)
	No		Yes				
	No	%	No	%			
Controls	66	74.2%	23	25.8%	0.991	Ref	Ref
Insulin cases	72	74.2%	25	25.8%		1.1 (.51-1.9)	1.0 (0.38-2.1)

## Discussion

Hirsutism is a condition in which females grow excessive body hair in androgen-dependent areas such as the lips, chin, chest, abdomen, back, and femoral region [13]. The prevalence of hirsutism worldwide was estimated to be 10% of the overall female population [14]. In this study where 186 diabetic females were studied, it was found that 25.8% of them had hirsutism (Figure 2). Such an increase in the number can be attributed to many factors. First, the elevated BMI since 37.1% of the participants were overweight and 38.2% were frankly obese. Such a hypothesis goes in line with what was found in an Iranian cross-sectional study, which stated that hirsutism is more commonly encountered in participants with high BMI (P > 0.001) [15]. However, in our study, we did not reach similar findings. Hirsutism was not significantly correlated with BMI (P = 0.132; Table 3). Moreover, another study examined women between 19 and 36 years of age who had various combinations of hirsutism, obesity, and/or oligomenorrhea, implying the same conclusion that obesity by itself and alone is not associated with hirsutism since the plasma androgen levels were normal among those who were only obese [16].

Age was found to be significantly associated with the development of hirsutism among age groups (<30, 30-49, and 50+; P = 0.049; Table 3). It seemed that the prevalence of hirsutism decreases as age advances; hence, the percentages of hirsutism among the groups were 37.5%, 30.8%, and 18.0%, respectively (Table 3). Such a conclusion is consistent with what was found in the literature. For instance, a Chinese large-scale, cross-sectional study that included 10,120 females revealed that as the age increases, the modified Ferriman-Gallwey score decreases [17]. A similar finding was found in another Chinese study where the prevalence of hirsutism among the age group of 41-45 years was only 1.5% while it reached 14.4% among those who were in the age group of 20-25 years [18].

Regarding the types of DM, it was discovered that the type of diabetes made no difference and had no significant correlation with the development of hirsutism (P = 0.469). Such a finding is attributable to the fact that patients with T1DM have insulin resistance as well the essence of the pathophysiology of T2DM [1,19].

Finally, to study the effect of insulin therapy on the development of hirsutism, a logistic regression analysis was conducted adjusted for age, type and duration of DM, PCOS, and BMI. It was found that insulin therapy does not have either a positive or negative effect on hirsutism as an outcome (OR = 1.0, 95% CI: 0.38-2.1; Table 4).

Our study has some limitations due to the lack of institutional support and funds, but we did everything possible to overcome them as possible. To obtain data with reasonable integrity and dependability, most of the participants were contacted in person at a diabetes center. Furthermore, to reach distant regions in the kingdom, some of the data were collected from WhatsApp groups of diabetic members.

While our findings were consistent with the literature and there were no surprises, the sample size that we could collect was small (186) and can hardly represent the Saudi population. Moreover, the convenience sampling technique, which was used, jeopardized the integrity of the data and made it susceptible to bias.

Future studies should take this study a step further and assess the effect of other drugs on hirsutism. A clustering sampling technique can also be considered to ensure the representativeness of the data.

## Conclusions

Many factors were examined to reveal their associations with hirsutism in diabetic females. Neither the type of diabetes nor insulin intake was significantly correlated with the development of hirsutism. On the other hand, age was found to be significantly associated with the development of hirsutism among age groups (<30, 30-49, and 50+; P = 0.049). It seemed that the prevalence of hirsutism decreases as age advances.
